# Characterizing Exposomes: Tools for Measuring Personal Environmental Exposures

**DOI:** 10.1289/ehp.120-a158

**Published:** 2012-04-01

**Authors:** Kellyn S. Betts

**Affiliations:** **Kellyn S. Betts** has written about environmental contaminants, hazards, and technology for solving environmental problems for publications including *EHP* and *Environmental Science & Technology* for more than a dozen years.


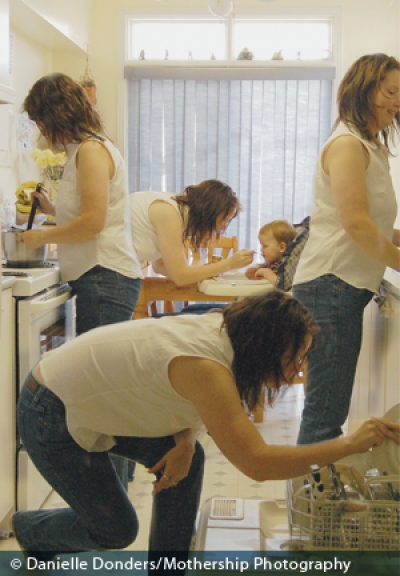
Exposures that cause chronic diseases usually take place years, perhaps decades, before disease is diagnosed. Biomarkers collected at single points of time therefore cannot tell the whole story of how disease occurs in an individual. For that, one must look to the “exposome,” or the compilation of exposures experienced over an individual’s lifetime. But efforts to link environmental exposures to disease have been stymied by the difficulty of accurately measuring those day-to-day exposures and the substances that are present in people’s bodies.

The term “exposome” was initially coined in 2005 by Christopher Wild,^1^ who now directs the International Agency for Research on Cancer, in recognition of the failure of genetic factors to explain most variability in human diseases. The exposome concept reflects the reality that people are exposed to potentially health-impairing agents from both pollution and nonpollution sources, including industrial chemicals, combustion emissions, radiation, heat/cold, noise, and food. The exposome also includes behavioral factors, such as activity levels and responses to stress. Finally, an individual’s exposome includes his or her microbiome,^2^ or vast personalized assembly of commensal microbes. All these exposures and factors can vary over the course of a day, not to mention over the weeks, months, and years that make up a lifetime.

A person’s exposome is the sum total of the many exposure factors that fill the days, months, and decades of that person’s lifetime—the exposures to chemicals, radiation, heat/cold, noise, food, stress, and other environmental agents; the health behaviors and activities; and the unique profile of commensal bacteria that make an individual an individual.

In the last few years, tools and methodologies have begun to emerge that hold promise for more easily capturing information about at least some of the environmental exposures that an individual may come into contact with over the course of his or her lifetime. The new tools come from a wide range of disciplines—some of which fall outside the usual domain of environmental health—and they are already helping researchers amass data on real-world exposures. These tools also hold promise for conducting studies that uncover unexpected links between environmental exposures and disease.

Several of the most promising tools and approaches were discussed at a workshop of the National Academies’ Emerging Science for Environmental Health Decisions committee in December 2011.^3^ Some of these tools are already helping researchers get a handle on how environ-mental factors contribute to important health risks, including cardiovascular disease and cancer, says Steve Rappaport, director of the Center for Exposure Biology at the University of California (UC), Berkeley, who organized the workshop.

## Measuring External Exposures

Tools for measuring the exposome are aimed at assessing exposures that take place both outside the body (the “exposure dose”) and inside (the “absorbed dose”); both are important for determining whether an environmental agent causes actual harm, says Linda Birnbaum, director of the National Institute of Environmental Health Sciences (NIEHS). For example, as some studies have shown, such as research involving measurements of arsenic in soil, house dust, and urine,^4^ a big increase in external exposure may not necessarily result in a major increase in internal exposure. At the same time, if you can’t tell where an internally measured chemical came from, it’s impossible to prevent the exposure.

Some of the new tools for measuring external exposures capitalize on the fact that the majority of the world’s citizenry—approximately 5.9 billion people—are cell phone subscribers.^5^ Cell phones already contain components that make them suitable for collecting key information associated with environmental exposures, points out Michael Jerrett, an associate professor in the Environmental Health Sciences program at the UC Berkeley School of Public Health. These instruments include ambient light meters, Global Positioning System sensors, and accelerometers, which measure movement. The latter two instruments can indicate when people travel by motor vehicle, which can be a major source of exposure to air pollutants, he says.

In an unpublished pilot study in Barcelona, Jerrett has been testing cell phones’ suitability for tracking environmental exposures. Students’ movements, as tracked by cell phones and other wearable devices, are overlaid on models developed by the city’s Energy Agency and others to predict air pollution levels. Jerrett says measurements collected via cell phones compare quite favorably with those taken by equipment that has traditionally been used to measure personal exposures, which was often the size of a backpack.

Another way that cell phones can help researchers is by interfacing with devices that collect important exposure-related information. One promising device is the Bluetooth-enabled SensPod monitor, which collects data on ozone, carbon monoxide, carbon dioxide, nitrogen oxides, noise, and ultraviolet radiation. In Copenhagen, Jerrett says, a network of individual cyclists travel through the city with SensPods mounted on their bikes. The monitors inform the cyclists about their personal exposures as they move through the city, and the data can be uploaded to an application that compiles them into a pollution map. Users can pair their SensPods with an Android smartphone via a mobile application that lets the two devices communicate and share data with the larger network of SensPod users. People in more than 20 countries in Europe, Asia, and North America are using the mobile sensors, according to Sensaris, the company that makes the devices.^6^ It’s no stretch to imagine investigators using these devices for research purposes.

Among the investigators working to expand the array of chemicals that can be detected by handheld sensors is Nongjian (NJ) Tao, director of the Center for Biosensor and Bioelectronics at the Arizona State University Biodesign Institute. He has created a wireless, wearable device the size of a cell phone that is capable of sensing petroleum-derived hydrocarbons, such as benzene, toluene, ethylene, and xylene (all of which are known or suspected human carcinogens^7^). Field-testing at an Arizona State waste management facility showed that the sensor could detect acid vapors associated with waste management, including phosphoric acid and hydrochloric acid. Tao says his devices have proven sufficiently sensitive to detect benzene, toluene, ethylene, and xylene at concentrations of 1 ppb, comparable to commercially available detectors.

**Figure:**
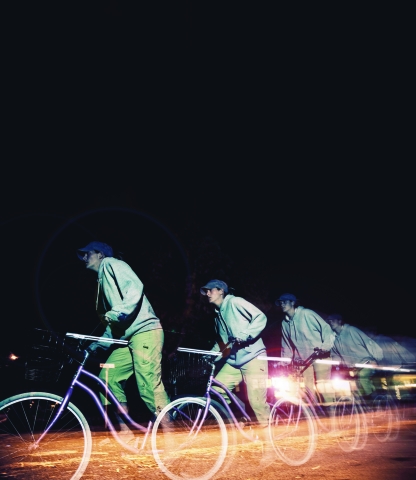
Physical exertion is an important consideration when measuring exposure, because activity levels can affect how much of a pollutant a person inhales. In one study of different travel modalities, people riding bicycles inhaled more than 8 times as much air per minute as people driving cars and half again as much air as people who walked.8 Of course, the answer to avoiding exposures is not to exercise less—rather, smart technology may someday advise travelers on small behavioral tweaks (such as falling behind the traffic ahead or taking a slightly different route) that could significantly reduce exposure to pollutants. P2 Photography

The tests Tao has conducted to date may be useful for evaluating personal exposures because they can generate results similar to those shown by U.S. Environmental Protection Agency monitoring systems. At the same time, the handheld devices can identify peaks the stationary monitors might miss. Tao is gearing up to begin pilot-testing the monitors in epidemiologic studies.

Another important aspect of personal exposure revolves around individuals’ levels of exertion. Stephen Intille, an associate professor in the College of Computer and Information Science and the Bouvé College of Health Sciences at Northeastern University, led the development of the Wockets system, a wearable device capable of recording people’s physical activity. Such data are important to exposure assessment because physical exertion can change the dose of pollution a person receives. In one study, people driving a car or riding in a bus inhaled about 4.5 L air per minute, whereas subway riders inhaled 10 L/min, people walking inhaled 23 L/min, and cyclists inhaled 37 L/min.^8^

Intille’s Wockets are different from consumer-targeted wearable activity monitors, such as heart-rate monitors and pedometers, in that they provide continual data on the type, intensity, duration, and location of the wearer’s upper- and lower-body physical activity for months at a time. They also collect data on compliance so that researchers know whether the monitors are being used. The Wockets were initially designed with input from a group of self-described “nontechnophile” volunteers aged 22 to 82 to ensure they are easy enough for even the least tech-savvy study participant to use. Intille’s team has also created “reminder” applications for Android phones and Windows mobile software to prompt participants to comply with research protocols. He hopes to have collected enough data to verify that the Wockets work as promised by the end of 2012.

Because the data from personal sensors such as the ones Sensaris produces can be posted online in near real time, it sets the stage for what Jerrett calls “participatory sensing networks” fed by inputs from wired citizens. (Although the Wockets data also are available very quickly, access to these data will be strictly controlled by the researchers, Intille stresses.) Whether the data come from individuals or centralized monitoring stations, they have great educational potential, Intille says—people participating in the network could learn about potential exposures associated with any given point in space and time, and having detailed data on exposures may also enable researchers to design interventions to reduce exposures, which could be programmed into smart devices.

“It’s one thing to know where people are exposed or how much people are exposed to, but once you know that as well as something about their behavior, you may be able to help them change their exposure levels,” Intille says. For example, smart technology might reveal when small changes in behavior (such as staying farther away from the cars ahead of you in traffic or walking a slightly different route) could effect significant changes in exposure to pollutants that exacerbate asthma, he points out. However, Intille and Jerrett agree that important privacy issues need to be worked out before these concepts can be fully realized.

## Internal Exposure Data

A major advantage of focusing on the internal exposome is that you don’t necessarily need to know exactly what you’re looking for in order to find something important to human health, Rappaport says. “By comparing complex patterns of chemical signals detected in the blood of healthy and diseased persons, it is possible to pinpoint particular chemicals whose levels are higher or lower in the people with disease,” he explains. This, he says, holds promise for helping scientists ferret out and characterize the heretofore unknown risk factors that underlie a large portion of the burden of chronic disease.

Technologies for collecting internal exposome data include efforts using blood plasma, urine, feces, and cells from inside one’s cheek or nostril. Some of these technologies already exist for other purposes. For example, Rajeshwari Sundaram, an investigator at the Eunice Kennedy Shriver National Institute of Child Health and Human Development, points out that over-the-counter fertility monitors used by couples seeking to become pregnant can be useful for collecting hormonal data from women of child-bearing age. Sundaram is involved in the National Institutes of Health’s Longitudinal Investigation of Fertility and the Environment (LIFE) study, which is using the monitors to capture daily changes in levels of reproductive hormones in a group of women who are trying to become pregnant. The LIFE study, which also involves men, is investigating how exposure to a variety of endocrine-disrupting compounds affects hormonally driven issues such as semen quality, time to pregnancy, infertility, pregnancy loss, gestation duration, and birth size.^9^

Another project under way to collect internal exposome data is headed up by Avrum Spira, a pulmonologist at Boston University. Spira is looking at gene-expression profiles in the human airway as signatures of internal exposure to smoke from tobacco and cooking fires. The group is currently focused on studying airway expression of the small noncoding RNA sequences known as microRNAs, or miRNAs, which regulate the genetic response to smoking.^10^ Spira’s group’s work is based on the hypothesis that cigarette smoke and other inhaled exposures alter epithelial cell gene expression throughout the respiratory tract^11^ and that variability in this gene-expression response is associated with risk for developing lung disease.^12^ One of the group’s ongoing projects is to identify novel miRNAs in the airway that may ultimately serve as biomarkers for detecting lung cancer based on a sample that can be easily captured through the nose or mouth. The team is also investigating whether exposure to burning biomass, such as through cooking fires, alters gene expression in these cells.

One of the most unexpected findings to result from an internal exposome investigation was published last year, when a group led by Stanley Hazen, head of the Cleveland Clinic’s Preventive Cardiology and Rehabilitation section, gained attention for identifying a potential role in cardiovascular disease risk played by consumption of choline and other nutrients in concert with the microbiome.^13^ According to Hazen, the microbiome is particularly important because it is a filter of what he calls our largest environmental exposure—what we eat—and is a major contributor to our internal exposure.

Hazen is the principal investigator in a clinical study that is following more than 10,000 patients in a bid to identify small molecules in blood plasma and related pathways that predict an increased risk for major cardiovascular events such as heart attacks. By studying samples from 150 randomly selected people who experienced a heart attack or stroke in the three years following enrollment, together with age- and sex-matched control subjects, Hazen’s group detected a host of candidate compounds associated with cardiovascular risk.

The investigation revealed that when animals and people consume diets rich in choline (a compound abundant in meat, poultry, and eggs), their gut microbes can transform the choline to trimethylamine. Trimethyl-amine is rapidly metabolized in the liver to trimethyl-amine *N*-oxide (TMAO). Hazen found that mice with higher TMAO levels had accelerated thickening of the artery walls due to accumulation of cholesterol, compared with mice with lower TMAO levels. Hazen’s group further demonstrated that a cocktail of broad-spectrum antibiotics could suppress intestinal flora in mice and prevent production of atherogenic TMAO from the choline in ingested egg yolk lecithin.^13^

**Figure:**
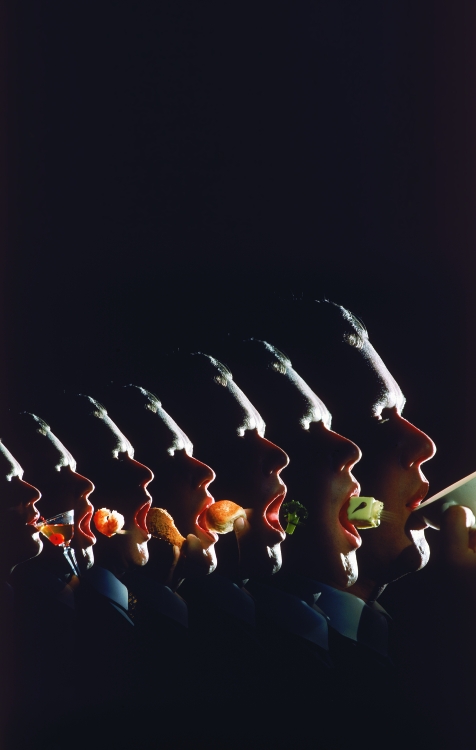
The microbiome is particularly important because it is a filter of perhaps our largest environmental exposure—our diet. Moreover, different intestinal bacteria can convert contaminants into new forms that may be more or less bioavailable than the original compound. Variations in individuals’ microbiomes could help explain why different people have different levels of susceptibility to environmentally influenced diseases. Ralph Crane

Hazen also reported that in a group of nearly 2,000 cardiovascular disease patients and controls, plasma TMAO levels predicted the future risk of cardiac events independent of traditional risk factors. ^13^ This suggests that a person’s microbiome profile could affect his or her heart-attack risk as much as or more than diet. It also could help explain why some people can get away with eating cholesterol-rich diets and others can’t—maybe those with gut flora that are poor at making TMAO are at less risk from eating high-fat diets, Hazen says. Although choline is an essential micronutrient crucial for brain development, many people may be getting too much of it, adds Hazen, in part because of the widespread use of lecithin in commercial baked goods to keep them soft and chewy.

There are at least 10 other examples where researchers have used an untargeted “omics” screening approach—such as that used by Hazen’s group—to identify potential markers of disease, Rappaport says. “By accumulating the biologically active chemicals from these studies in a library of potential environmental hazards, future investigators will be able to determine whether these chemicals are involved in a host of diseases whose origins are currently unknown,” he says.

## Managing the Data

To truly characterize the exposome, how-ever, these internal and external measurement modalities must be inte-grated. Although external exposures don’t lend themselves to the untargeted omics approach that has led to recent advances involving the internal exposome, Rappaport stresses that air and water pollution and other external factors, such as exercise and stress, contribute to human diseases and must be controlled. “This will require more and better methods for simultaneously monitoring multiple targeted external stressors and, in time, for combining external measurements with internal exposomes,” he says.

The ability to compare samples taken before and after any manifestations of disease are present is an obvious advantage to studying the exposome. Rappaport says investigators can move the science forward by developing prospective cohort studies that collect data on external stressors while also obtaining and storing blood or other biospecimens for future measurements of internal exposomes.

Accordingly, Nathaniel Rothman, head of molecular studies at the National Cancer Institute, says the 40–50 general prospective cohort studies currently under way throughout the world have a variety of biological samples and history information available that scientists may be able to use in future exposome studies. Studies where repeat samples have been taken may prove especially useful, he notes. Birnbaum adds that the NIEHS maintains a huge library of biological specimens from studies conducted by intramural investigators. Suzanne Fitzpatrick, senior science advisor in the Office of the Chief Scientist at the Food and Drug Administration, points out that the samples collected during drug trials may be available for use by other researchers, too. Paul Elliott, chairman of epidemiology and public health medicine at Imperial College London School of Public Health, says the United Kingdom is considering a proposal to repurpose the facilities built for drug testing in the 2012 Summer Olympic Games to invest in what he called “exposomic” research.

Chirag Patel, a postdoctoral research fellow at the Stanford University School of Medicine, thinks the comprehensive connection of environmental factors to disease is now possible using the high-throughput analysis methods common in genome-based investigations. His proof of concept for such so-called environment-wide association studies used blood serum and urine samples from the National Health and Nutrition Examination Survey (NHANES) cohorts from 1999 through 2006. In 2010 his group reported unexpected associations between type 2 diabetes and environ-mental exposures to heptachlor epoxide and ©-tocopherol.^14^ They also found associations with polychlorinated biphenyls (PCBs)—which have previously been linked to this form of diabetes—and with pesticides. Investigators elsewhere have hypothesized that these chemicals might increase risk of obesity and metabolic diseases.

More recently, Patel’s group used the same techniques with NHANES data to screen for associations between environmental chemicals and blood lipids.^15^ The preliminary findings suggest that higher levels of triglycerides and lower levels of “good” high-density lipoprotein cholesterol may be linked with higher concentrations of fat-soluble contaminants such as PCBs and dibenzofurans. Patel says these associations merit more investigation, although he also makes it clear that the potential for confounding and reverse causal biases needs to be investigated via longitudinal and followup studies. That is, because the studies are cross-sectional in nature, it is entirely possible that the associations are a consequence of disease rather than a cause.

In the longer-term future, Patel envisions a time when improvements in our ability to measure both the internal and external exposomes will enable investigators to assess hundreds to thousands of different factors in connection to specific diseases or health states. To use that information to discover associations with disease, he foresees that new analytical and informatics methods will be required. This was an issue in early genomics studies, and it eventually led to a proliferation of new statistical techniques and the field of bioinformatics, he points out.

Birnbaum, for one, is cautiously optimistic about the promise of environment-wide association studies. “Genetic factors are inherently less complicated than environ-mental factors,” she stresses. “We may need some additional tools to deal with the environment. While bioinformatics is doing a great job now with the genetic information, I think we have a long way to go, and we need a lot more bioinformatics approaches and understanding to deal with the wealth of information that will come from the exposome.”
